# Barriers underlying care gaps in Singapore’s mental health landscape and suggestions for improvement from service providers’ perspectives: a qualitative approach

**DOI:** 10.3389/fpubh.2025.1527521

**Published:** 2025-05-19

**Authors:** Siang Joo Seah, Charlotte Chao Min Tan, Mary Su-Lynn Chew, Jin Jin Lim, Dhiya Mahirah, Yi-Ching Lynn Ho, Sing Ai Lee, Ee-Lian Lee, Sungwon Yoon, Vicknesan Marimuttu, Ngar Yee Poon, Julian Thumboo

**Affiliations:** ^1^Centre for Population Health Research and Implementation, Regional Health System, Singapore Health Services Pte Ltd., Singapore, Singapore; ^2^Bridgepoint Health, Singapore, Singapore; ^3^PsyMed Consultants, Singapore, Singapore; ^4^Duke-NUS Medical School, Singapore, Singapore; ^5^Department of Psychological Medicine, KK Women’s and Children’s Hospital, Singapore, Singapore; ^6^Health Services Research Unit, Singapore General Hospital, Singapore, Singapore

**Keywords:** mental health, care gaps, service providers’ perspectives, suggestions for improvement, qualitative

## Abstract

**Background:**

Despite the high prevalence of mental health conditions globally, there are few studies investigating perspectives of providers on barriers underlying care gaps. Therefore, the primary aim of this project was to understand barriers underlying care gaps and suggestions for improvement from mental healthcare service providers in Singapore.

**Methods:**

Semi-structured interviews were conducted with 20 mental healthcare providers from public and private sectors, including administrators, practitioners, and an advocate. Participants were recruited via purposive sampling and snowballing. Interview transcripts were analyzed using thematic analysis.

**Results:**

Barriers underlying service accessibility included insufficient integration across organizations and sectors for timely referrals and concerns about stigma of receiving services. Barriers to service effectiveness included limited public sector bandwidth and lack of supervision requirements for private sector allied health practitioners. General practitioners (GPs) faced financial and referral issues when serving as first touchpoint for mental healthcare. Fragmentation of information systems hindered coordinated care implementation, while lengthy referral forms and complex referral routes created additional obstacles. Public sector manpower issues and heterogeneity in frontline staff’s skills for right-siting patients hindered capacity maintenance.

**Conclusion:**

These findings highlight systemic challenges related to under-resourcing and a lack of coordination across sectors and organizations, ultimately hindering equitable access to mental healthcare. To address these challenges, recommendations include expanding insurance coverage, strengthening private sector regulation, and reforming reimbursement structures for integrated care. Streamlining referrals and improving data sharing will enhance coordination. Additionally, normalizing mental health conversations, empowering support networks, and increasing access to community-based services will reduce stigma and provide timely care, ultimately improving service delivery and access.

## 1 Introduction

Despite the high prevalence of mental health conditions globally (1, 2), treatment gaps have persisted (2–4), contributing to an increase in the global disease burden (1, 2). For example, in Singapore, the prevalence of poor mental health has increased from 13.4% in 2020 to 17% in 2022 (5). Despite ongoing efforts to improve mental healthcare, treatment gaps have remained high in Singapore (4). The overall treatment gap for any mental health condition, including mood disorders, anxiety disorders and alcohol use disorders was 78.6% (4). The treatment gap for a condition was calculated by taking the difference between the prevalence of the condition in the past 12 months and those who had received treatment for it, representing the percentage of individuals who needed treatment but did not receive it (4). Unsurprisingly, mental health conditions were among the top five contributing causes to the Singapore population’s disease burden (6).

Pathare et al. (7) introduced the concept of mental health care gaps which, compared to treatment gaps, facilitates a comprehensive understanding of unmet needs among those with mental health conditions. Besides treatment gaps, which connote a lack of biomedical and clinical treatments, care gaps encompass gaps in psychosocial interventions for mental health conditions, and gaps in healthcare for physical comorbidities, which individuals with severe mental health conditions have a higher risk of (7–9). In Singapore, the focus of mental healthcare policy has shifted from acute institutionalized care to community-based care supported by hospital specialists (10, 11). This policy shift entails ongoing efforts towards the integration of health and social care to support individuals with mental health conditions (12), and is in line with the World Health Organization’s guidelines for mental healthcare’s focus to transition to community-based care that is accessible, equitable and stigma-free (13). As part of the ecosystem for community-based care, private sector general practitioners (GPs) and public sector polyclinics, which are state-run clinics that provide subsidized primary care, can facilitate continuity of care for discharged patients whose conditions have stabilized (11, 14).

There have been relatively fewer studies conducted to understand barriers underlying mental health care gaps. Among studies conducted, users’ cost concerns and perceived stigma related to treatment were factors contributing to treatment gaps in Singapore and the United States (4, 15). Inadequate knowledge of types of services for different conditions and corresponding service costs, coupled with concerns about how others might perceive them for receiving treatment, make those with mental health conditions hesitate to seek help (4). Other barriers in Singapore and Japan included low mental health literacy and insufficient knowledge about appropriate avenues to seek help (3, 4). As these studies illustrate barriers from users’ perspectives, complementary studies conducted to understand challenges from providers’ perspectives are also needed. Such studies can shed light on how barriers to care gaps could be addressed by policy planning and adjustments to service and program implementation.

Barriers to care gaps highlighted by providers in the limited literature include capacity issues, inefficient care pathways hindering the integration of mental health into healthcare settings, primary care providers’ insufficient knowledge and unwillingness to provide mental healthcare, and lack of communication between health professionals (16–18). Capacity issues included challenges retaining trained staff due to perceived lack of career progression and dissatisfaction with remuneration (17). Increase in provider burnout during the pandemic was a challenge facing mental healthcare (19). Staff burnout contributes to turnover (20) and is negatively associated with patient satisfaction with mental health services (21), worsening care gaps in care quality. Inefficient care pathways contributed to challenges in providing referrals (16). Although primary care plays a crucial role as the first touchpoint and in continuity of care, some primary care providers might be reluctant to provide mental healthcare due to concerns of lacking adequate education to manage mental health conditions (18).

There are still gaps in the literature to be addressed for a holistic and in-depth understanding of barriers to the provision of accessible, effective, and sustainable mental healthcare. As the private sector plays an important role in the provision of mental healthcare in Singapore and internationally, the first gap is the lack of studies which explicitly included service providers from the private sector. Doing so would facilitate a cohesive understanding of challenges from the private and public sectors that would have a collective impact on service users with heterogenous needs. The second gap is the relative lack of studies that included perspectives of providers from a wide array of professional roles, including administrators and mental health advocates. With their experiences in providing directions to address operational issues such as inadequate manpower and providing professional inputs to shape national-level mental health initiatives, administrators could provide insights into operational and policy-level challenges. With their roles in facilitating dialogue on reducing mental health-related stigma and having more support for mental health, advocates could provide their insights on broader socio-cultural issues that contribute to care gaps and how resources within one’s social networks, such as family members and friends, can be better harnessed. To address these gaps in the literature, we conducted a service evaluation of the mental healthcare landscape in Singapore, with the aim of understanding barriers underlying care gaps and suggestions for improvements, from the perspectives of local mental healthcare service providers. As the risk of comorbidity increases with a mental health disorder (22), and the overall treatment gap in Singapore is high, the scope of our evaluation was on the mental healthcare system and factors influencing the extent to which it addresses the population’s mental health needs, instead of focusing on a single condition.

## 2 Materials and methods

A qualitative approach was adopted using the Reach, Effectiveness, Adoption, Implementation and Maintenance (RE-AIM) (23) and Sustainable integrated chronic care models for multi-morbidity: delivery, Financing and performance (SELFIE) (24) frameworks, to evaluate mental healthcare services in Singapore. The qualitative evaluation was guided by the Consolidated criteria for Reporting Qualitative research (COREQ) Checklist where applicable.

### 2.1 Theoretical frameworks

The RE-AIM and SELFIE theoretical frameworks were used to design the interview guide and to guide thematic analysis of the data. Although RE-AIM has traditionally been used to evaluate a program or intervention, Holtrop et al. posited that it could be expanded to evaluate a policy (25). Hence, we have used RE-AIM to systematically explore barriers related to *reach, effectiveness, adoption, implementation and maintenance* of mental healthcare services shaped by Singapore’s policy emphasis on community-based care augmented by hospital specialists (25). This framework provided a structured lens to examine how services operate and the challenges faced by local providers in reducing care gaps and fulfilling ideal stakeholder roles.

Complementarily, the SELFIE framework was used to provide a comprehensive understanding of the contextual factors influencing service delivery across micro, meso and macro levels (24). Recognizing that individuals with mental health needs also experience multi-morbidity and complex needs (26), the SELFIE framework allowed us to situate identified barriers within individual-level and broader system-level influences. Specifically, the framework’s micro, meso, and macro levels were applied to analyze and categorize barriers related to reach, effectiveness, adoption, implementation and maintenance. For example, to understand barriers underlying reach of services, we considered micro-level factors (e.g., users’ financial constraints and service preferences), meso-level factors (e.g., business models of private providers), and macro-level factors (e.g., healthcare financing policies) (24). This multi-level analysis, facilitated by SELFIE, allowed us to examine the interconnectedness of these barriers and identify actionable recommendations for improvement in implementation and practice to address these barriers at each level.

### 2.2 Setting

In Singapore, approximately 1800 private sector GP clinics cater to approximately 80% of the demand for primary care (27), with over 400 GPs trained by specialist-led teams to provide mental health care (28). For individuals requiring higher intensity of care, public hospitals provide both inpatient and outpatient psychiatric services (11). Outpatient private sector psychiatric services are also available. Approximately one in five registered psychiatrists are from the private sector (5, 29). Working alongside to support practitioners are the health and social care administrators who facilitate efficient service implementation through their work in areas including human resources, finance, operations, communications, and organizational development (30–32).

### 2.3 Sampling and recruitment

Participants were recruited using purposive and snowball sampling to ensure representation across various levels of the mental healthcare system (33). Purposive sampling targeted providers in diverse roles including practitioners (e.g., psychiatrist, psychologist, family therapist), administrators, and an advocate. Representation from various settings (community, primary and tertiary care) across both public and private sectors was sought to explore potential differences and commonalities in barriers and opportunities across diverse contexts. Potential participants were identified through professional organizations and co-authors’ existing networks. Two co-authors (SAL and ELL) compiled the initial list of potential participants based on these criteria. The participant who was recruited via snowballing was recommended by one of the potential participants. Among the 26 potential participants who were invited, 20 agreed to participate in the project, while five did not respond to the invitation and one declined as he recommended his colleague as a more suitable participant.

The team invited potential participants via email to participate in an interview approximately 1 h in duration and arranged for an interview session with each potential participant who agreed. At the start of each session, the aims of the project were explained and verbal informed consent was obtained from each participant by a project team member before proceeding with the interview. To ensure confidentiality, each participant was assigned a unique identification code.

### 2.4 Data collection

The semi-structured in-depth interviews were conducted online or in person, according to participants’ preferences. Topics of discussion for the interview guide included service users’ demographic profiles, underserved population segments, effectiveness of existing services, adoption of mental healthcare models and ideal roles for various stakeholders, level of integration of services, sufficiency of mental healthcare capacity, and broader socio-cultural issues affecting mental healthcare (Please see Supplementary file 1 for the interview guide).

To ensure consistency among the interviewers, all interviewers (CCMT, MSLC, JJL) were trained by the first author, SJS, who has experience in conducting semi-structured interviews and was also one of the interviewers for this project. The interviews were conducted in English between March to May 2023 via Zoom, an online platform. All interview sessions were audio-recorded and transcribed verbatim. Notes were also taken during the interview sessions. Interviews ranged from approximately 38 min to 1 h and 27 min, with a mean duration of approximately 1 h and 3 min. Data saturation was reached for this study as the interviews provided sufficient data for the project team to understand underlying barriers pertaining to each of the *a priori* domains defined by the RE-AIM framework and the relevant domains within the SELFIE framework, such as policies and practices to integrate care for service delivery, educational and workforce planning for workforce capacity, financing care, and considerations underlying access to information (34). This approach to determining data saturation and sample size adequacy is in line with Braun and Clarke’s posit of limitless possibilities for interpretation in a dataset and that researchers would need to make an interpretative judgement of the point at which the analysis is sufficient for the theoretical framework (s) in use (35). All participants who completed the interview were each offered reimbursement as a token of appreciation.

### 2.5 Ethics statement

This project received an exemption from review and approval was not required from the SingHealth Centralised Institutional Review Board (CIRB) as CIRB considered it to be a service evaluation project.

### 2.6 Data analysis

NVivo (release 1.7.1) was used to organize and manage the data from the transcripts. The thematic analysis of the data followed a hybrid approach of deductive and inductive coding for development of themes and subthemes (36). This allowed the team to harness the rich complexity within the data provided by participants with heterogenous professional profiles via inductive analysis, while allowing for a structured analysis of the data using the well-established RE-AIM implementation science framework via deductive analysis (37). We followed a six-step approach for data analysis (37, 38). The first step was the familiarization of the transcripts to have a more in-depth understanding of the data. The second step was line-by-line coding to generate initial codes. Deductive analysis was done to categorize the data using *a priori* codes under the broad categories of Reach, Effectiveness, Adoption, Implementation and Maintenance. This was supplemented by inductive analysis via open coding of data related to local mental healthcare to provide elaboration as subthemes for the a priori codes. To facilitate inter-coder reliability, selected transcripts were independently coded by two separate coders, the second author CCMT and the third author MSLC, and any disagreements in coding were resolved in discussion with the first author SJS. The third step was the grouping of inductive codes into meaningful sub-themes, using the SELFIE framework where relevant. Codes were collapsed if needed. The fourth step was to review the themes and subthemes. The fifth step involved refining themes and subthemes through various iterations of discussion and renaming them if needed. The sixth step was the selection of relevant quotes to elaborate on the themes and subthemes.

To ensure the trustworthiness of this qualitative study, we adhered to the criteria outlined by Lincoln and Guba (39). Credibility was enhanced through purposive and snowball sampling strategies, aiming to recruit information-rich participants representing diverse roles and settings across both public and private sectors. Dependability was addressed by maintaining a detailed audit trail of the data collection and analysis process, including audio recordings, verbatim transcriptions of interviews, and researcher notes. Confirmability was promoted through reflexivity, with researchers critically examining their biases through a regular peer debriefing process. Furthermore, two researchers (CCMT and MSLC) independently coded selected transcripts, and any discrepancies were resolved through discussion with a third researcher (SJS), enhancing the consistency and objectivity of the analysis.

## 3 Results

A sample of twenty participants were recruited and completed the interview. Among the participants, 11 were from the public sector and nine were from the private sector. Participants who were administrators included those in senior management of their organizations. Those who were practitioners included GPs, psychiatrists, psychologists, and a family therapist. Participants who had roles in both public and private sectors were categorized based on their primary role. Table 1 presents a summary of participants’ profiles.

**Table 1:** Summary of participants’ demographic profiles.

Participant ID	Gender	Work sector	Areas of care	Job role/ Title	Job type
ID001	Female	Public	Community	Psychologist	Practitioner
ID002	Male	Public	Tertiary	Senior Management	Administrator
ID003	Male	Public	Tertiary	Psychiatrist	Practitioner
ID004	Male	Private	Primary	General Practitioner	Administrator
ID005	Male	Public	Tertiary	Senior Management	Administrator
ID006	Male	Private	Primary	General Practitioner	Administrator-Practitioner (practitioner role 50% of time)
ID007	Male	Public	Tertiary	Senior Management	Administrator
ID008	Male	Public	Tertiary	Senior Management	Administrator
ID009	Male	Private	Primary	General Practitioner	Administrator
ID010	Male	Public	Community	Senior Management	Administrator
ID011	Male	Private	Community	Psychiatrist	Practitioner
ID012	Female	Public	Community	Ambassador	Advocate
ID013	Male	Private	Tertiary	Psychiatrist	Practitioner
ID014	Female	Private	Community	Senior Management	Administrator
ID015	Female	Public	Community	Senior Management	Administrator
ID016	Male	Private	Community	Psychiatrist	Practitioner
ID017	Female	Public	Tertiary	Psychiatrist	Practitioner
ID018	Male	Public	Tertiary	Psychiatrist	Practitioner
ID019	Female	Private	Community	Family Therapist	Practitioner
ID020	Female	Private	Community	Psychologist	Practitioner

The findings are presented by RE-AIM domains to highlight key barriers to mental health care services. Table 2 summarizes these barriers by the domains. To contextualize these barriers within broader system dynamics, Table 3 maps them onto the micro, meso, and macro-level factors of the SELFIE framework and provides a high-level overview of corresponding recommendations. These recommendations are further elaborated in the *Implications of Findings* section.

**Table 2:** Barriers to mental healthcare services organized by the RE-AIM framework.

RE-AIM domain	Identified barriers
Reach (Ability to engage the target population)	Fragmented information exchange between healthcare and social sectors hindered timely referrals for individuals with complex needs Inadequate integration of mental health services with physical and developmental care contributed to delayed diagnosis and treatment Stigma surrounding mental health conditions deterred help-seeking, particularly among working adults and minors Financial constraints, including long wait times for subsidized care and limited insurance coverage, restricted access to services.
Effectiveness (Impact of services on intended outcomes)	Limited bandwidth in the public sector due to capacity constraints Lack of clinical supervision protocols in the private sector affecting care quality
Adoption (Uptake of mental healthcare models and stakeholder roles)	Financial viability challenges for GPs in adopting integrated care models Challenges in establishing multi-stakeholder collaborations
Implementation (Delivery of services as intended)	Fragmentation of information systems leading to disjointed communication across organizations Complex referral protocols creating coordination challenges
Maintenance (Sustained service delivery over time)	Workforce capacity and retention issues, including specialist shortages and high attrition rates Variation in frontline staff skills, affecting patient right-siting

System-level mapping of these barriers is elaborated in Table 3 using the SELFIE framework.

**Table 3:** Thematic mapping of findings using the SELFIE Framework (Micro / Meso /Macro levels).

SELFIE Component	Level	Barriers	Recommendations
Social and community support	Micro	Low mental health literacy and stigma hinder help-seeking.	Strengthen public education, awareness and peer support systems
Service delivery	Meso	Poor inter-organizational communication Disjointed referral processes	Standardize referral pathways and improve coordination between providers
Information and research	Meso/Macro	Fragmented information systems Poor integration of information between healthcare and social care sectors	Integrate digital health records across sectors and support care continuity
Workforce	Meso/Macro	Skill gaps and variable competencies among frontline staff High attrition rates and shortages of mental health specialists	Invest in training, staff support and workforce retention strategies
Financial arrangements	Macro	Inadequate insurance and financial support GP remuneration challenges limiting participation	Expand insurance coverage and reform financing to improve access and provider participation

### 3.1 Reach

Several barriers impacted the reach of mental healthcare services to engage individuals in need, particularly those with complex needs, financial difficulties or for whom stigma created a disproportionate barrier to help-seeking, such as working adults and minors. These barriers include fragmented information exchange, inadequate service integration, stigma around mental health, and financial constraints limiting access.

*Fragmented information exchange* between organizations and across the health and social care sectors was a barrier affecting timely access to services. Individuals with mental health needs often present with multimorbidity and complex circumstances that require coordinated care and information sharing. However, limited systemic communication and updates between providers constrained the ability to arrange timely referrals, especially for private sector practitioners lacking multidisciplinary team support. Even within the public sector, service providers faced challenges navigating an evolving landscape of services. As one practitioner described:

… one Centre might do autism assessments today and then tomorrow it don’t do it anymore. So the goalpost keeps changing … new services are coming up … knowing the map of mental health is even difficult for professionals. (ID003, Public practitioner)

The lack of clarity on service availability further compounded difficulties in coordination, resulting in delays in access. This fragmentation reflects a meso-level organizational and structural integration issue that maps onto the service delivery component of the SELFIE framework.

*Inadequate service integration* for physical, developmental, and mental health conditions further compounded these challenges, potentially leading to missed opportunities for early detection and treatment of mental health issues. Individuals with complex needs — including those whose mental health challenges manifested as relational issues that brought them to the attention of social service agencies or the police, as well as those who attempted suicide but were not injured—were particularly vulnerable to falling through the cracks when health and social care services were not well coordinated.

*Stigma related to mental health* emerged as another significant barrier limiting the reach of services. This was particularly pronounced for working adults and minors. Self-stigma often delayed help-seeking, while fears of disclosure deterred employees from accessing services like Employee Assistance Programs due to concerns about potentially compromising career development. Minors under 18 years old, for whom parental consent is mandatory to receive services, might experience care gaps when their parents did not provide the required consent due to concerns about the potential stigma that their child might experience from having utilized mental healthcare services. A lack of knowledge about mental health conditions and appropriate services for various types of mental health issues compounded this issue of stigma. One practitioner explained:

… they [parents] feel that … “Is it possible maybe my kid is just going through a phase? Maybe watch and wait?… I don’t want my kids on medications. So I know my kids is … having to a record that they saw someone”– that’s a big one. So … I don’t think it’s as simple as stigma … okay, if I was to tell a family that “Look … you don’t need to have to see a mental health psychiatrist or a psychologist, but you know what you can see? Your family doctor …” I think they’ll be more open to it. (ID003, Public practitioner).

*Financial barriers* also constrained access to care, particularly for those reliant on subsidized public sector services. Long wait times for subsidized care disproportionately affected individuals with limited financial means, while inadequate insurance coverage increased out-of-pocket costs and limited access to private sector services.

### 3.2 Effectiveness

Several barriers impacted the effectiveness of mental healthcare services in achieving meaningful improvements in clinical, social, and occupational outcomes. These barriers included limited public sector bandwidth, lack of supervision for private sector allied health professionals (AHPs), and challenges in ensuring consistent quality of service across different settings.

The evaluation of effectiveness is complex due to the absence of standardized outcome measures across providers. While practitioners holistically assessed client improvement through biopsychosocial indicators and family feedback, administrators emphasized process outcomes such as wait times for first and follow-up appointments.

*Limited provider bandwidth* in the public sector contributed to longer wait times for follow-up appointments, which in turn might adversely affect the rigour of treatment protocols and continuity of care, ultimately influencing the effectiveness of care received by patients:

… the frequency in which they can follow up is also quite long. So for example, most guidelines will recommend a psychotherapy on a weekly basis every week, but you don't see that happening in public hospitals. It's just because … they can't cope with the load. (ID018, Public practitioner)

For private sector providers, the *lack of mandatory supervision for AHPs* resulted in variability in service quality. This inconsistency poses challenges for patients seeking effective care, as navigating services and finding qualified providers becomes more difficult. As noted by a private administrator:

… mental health professionals are not mandated to continuously have supervision … and that is actually very important for them to be good at their job … so while it is not mandatory outside, within [ID014’s organization] it is. (ID014, Private administrator).

### 3.3 Adoption

The uptake of mental healthcare models and interventions by providers and organizations plays a critical role in shaping the availability of integrated care for individuals in need. A key approach highlighted by participants was the adoption of biopsychosocial models of care, which combined medical treatment, mental well-being services, and support for social issues. Providers with in-house multidisciplinary teams were better positioned to deliver such care, while others relied on inter-organizational referrals. This collaboration across organizations represents a meso-level organizational integration factor that maps onto the service delivery component of the SELFIE framework.

However, several participants—primarily from the private sector—reported not adopting any specific mental healthcare models in their practice. Across both public and private sectors, GPs were consistently identified as ideal first touchpoints for mental healthcare. Positioning GPs to triage and manage mild to moderate cases was viewed as a promising model to improve accessibility, reduce stigma, and provide holistic care—especially for individuals with co-occurring physical and mental health conditions. GPs could also support longer-term management of stabilized cases within the community through services such as medication prescription. Despite the promise of this model, barriers to adoption were significant.

*Financial viability* emerged as a major constraint, particularly in private practice settings. The time-intensive nature of mental healthcare consultations, combined with high operational costs, made it financially unsustainable for GPs without additional support or funding:

It's not financially viable to do. Even if I charge a proper consult, $40, $50, and I need 45 minutes, it's very emotionally taxing … the doctor costs is about … $100, $100 plus an hour. If you have three staff in your clinic, it's $20 per hour. For rental, it is about $10, $20K a month. If you spend one hour with the fellow, half an hour with the patient, or with the other staff, you need to bill $100 plus. The patient cannot afford. (ID006, Private administrator and practitioner).

*Limited access to AHPs* reflects a lack of effective collaboration between GPs and other service providers, creating a barrier to the adoption of integrated mental healthcare models. The resulting long referral wait times makes it challenging for GPs to effectively address patients’ mental health needs:

GPs don’t really typically work with psychologists or…counsellors. So they [patients] always get very upset when you know, the waiting time [for referrals to a psychologist or counsellor] is two weeks, three weeks … And… we don’t have the bandwidth to maintain these kind of services … I think there’s a short [shortage] of…good referral pathways…a very organised manner where we manage mild to moderate patients … within the community. (ID004, Private administrator).

These barriers, rooted in business case consideration and resource limitations, map onto the macro-level financing component and the meso-level service delivery component, respectively, of the SELFIE framework. It highlights the need for targeted funding and structural support to enable GPs to adopt and sustain mental healthcare delivery effectively.

### 3.4 Implementation

The effective delivery of mental healthcare services hinges on systems, processes, and protocols that ensure timely and coordinated care. Several barriers hindered the implementation of services, particularly for individuals with complex healthcare and social needs.

*Fragmentation of information systems* across healthcare and social service sectors hampered efforts to coordinate care plans. Social service agencies often lacked access to updated information on clients’ medical treatments, while healthcare providers might not be informed of community services patients were receiving. This created gaps in information exchange that disrupted continuity of care and increased the risk of individuals falling through service cracks. Underlying this fragmentation were organizational concerns about data ownership and patient confidentiality, which map onto the meso-level Information and Research component of the SELFIE framework.

*Fixed referral routes and administrative processes* delayed the timely implementation of services. Both providers and users faced challenges navigating fixed referral routes and cumbersome documentation requirements. Lengthy referral forms, additional assessments, and procedural bottlenecks posed significant hurdles, particularly for individuals already struggling with mental health conditions:

… a lot of times … it involves filling up lengthy referral forms, you may have to see another healthcare professional for referral before you actually access these services … to somebody who is suffering from a mental health disorder … it’s going to be very challenging to navigate. (ID009, Private administrator).

### 3.5 Maintenance

Several barriers constrained the maintenance of sustainable and effective mental healthcare services. Limited public sector manpower contributed significantly to long appointment wait times, and there was a need to standardize frontline staff’s competencies, particularly in terms of right-siting patients to the appropriate levels of care.

*Workforce shortages*, particularly the lack of psychiatrists, AHPs, especially clinical psychologists, and niche roles such as psychiatric nurses and psychiatric rehabilitation practitioners, present a critical barrier to sustaining accessible and effective care. This shortage contributed to limited public sector capacity and high attrition rates, particularly towards the private sector. This barrier can be viewed as a macro-level issue linked to workforce planning and education, in alignment with the SELFIE framework’s educational and workforce components. The workforce shortage has been further exacerbated by challenges in retaining talent within the public sector:

… what we really lack is allied health pipelines, particularly psychologists, or clinical psychologist, … and there's a large number of clinical psychologists in private practice, … there's a huge exodus following the pandemic. (ID002, Public administrator).

The high attrition to the private sector was due to various reasons, including high caseloads, insufficient time to provide quality care, and insufficient remuneration.

*Variation in frontline staff competencies*, particularly in the assessment of mental health conditions for right-siting clients, poses another significant barrier. When frontline staff lacked the necessary skills to assess and refer appropriately, individuals who did not require tertiary-level care were often inappropriately referred to hospitals, exacerbating the strain on already overburdened services:

… I don’t feel that there is a level of … competency in terms of assessment in front liners … What will happen is that everyone that says mental health will then be referred to a hospital or another centre. And you would inundate that center … I wanted to add that actually it’s not everyone doesn’t have competency, I think it’s very heterogeneous. (ID003, Public practitioner).

## 4 Discussion

Our study explored barriers contributing to mental health care gaps in Singapore, drawing on the perspectives of local service providers. We identified interconnected barriers at the micro (individual and community), meso (organizational) and macro (systemic) levels, mapped across the RE-AIM framework (23). These barriers span from challenges in reaching individuals to ensuring service quality and continuity, with workforce capacity and system integration emerging as persistent constraints. Stigma, public awareness, and financial constraints limit access, while workforce shortages, varying competencies, and fragmented systems undermine service effectiveness and sustainability. Our findings also highlighted the intersection of public and private sector challenges, providing new insights into the private sector’s struggles with financial sustainability, regulatory gaps, and referral bottlenecks in delivering mental healthcare.

At the micro level, our findings highlight low mental health literacy and stigma as significant barriers to early help-seeking. Self-stigma, perceived structural stigma, and parents’ limited understanding of mental health, were cited as factors that delay individuals – particularly children and adolescents – from accessing timely care. These findings corroborated Gunasekaran et al.’s findings which emphasized healthcare professionals’ concerns about how self-stigma, parents’ low mental health literacy, and structural stigma contributed to treatment delays (40). While nationwide guidelines in Singapore discourage the declaration of mental health conditions in job applications (41), lingering perceptions of structural stigma may still deter individuals from accessing workplace mental health services, out of concern for potential repercussions for their career progression. These findings underscore the need for ongoing efforts to reduce self-stigma and perceived structural stigma while strengthening mental health literacy within the community.

At the meso level, organizational challenges emerged as critical barriers to effective care delivery. Poor inter-organizational communication and disjointed referral processes contribute to fragmented care, delaying patient care and compromising service quality (42). Extending Qureshi et al.’s findings on inefficient care pathways (16), our study highlights how poor inter-organizational and inter-sectoral information exchange hampers timely referrals and coordinated care, especially for individuals with complex needs. Fragmented information systems and poor integration between healthcare and social care sectors exacerbated these challenges, contributing to delays in diagnosis and disruptions in continuity of care.

Workforce issues compound these structural barriers. High attrition rates in the public sector and variable competencies among frontline staff impede the delivery of quality care. Similar to Ross et al.’s findings that primary care providers expressed concerns about their competency in delivering mental healthcare (18), our study found heterogeneity in skills among frontline staff, particularly in the initial assessment and right-siting of patients. Furthermore, the lack of regulation regarding clinical supervision requirements for private sector allied health professionals (AHPs) contributes to inconsistencies in service quality making it harder for individuals to identify qualified providers. These issues underscore the need for improved training, clinical supervision and retention strategies to reduce variability in service quality across providers. Our findings also align with past studies reporting stigma-related barriers within the mental health workforce itself. Mental health professionals reported being discouraged from entering psychiatry during medical training while psychiatric nurses faced moderate to high associative stigma (43, 44) Such factors further constrain workforce sustainability and exacerbate service delivery challenges.

At the macro level, our study highlights how inadequate insurance coverage and financial support restrict access to mental healthcare. Financial arrangements, particularly the lack of sustainable remuneration models for private sector GPs, emerged as significant systemic barriers. Private GPs face additional challenges related to financial viability and limited referral pathways (45, 46). These factors must be addressed to strengthen the role of GPs as accessible first points of contact for mental health concerns. Furthermore, the lack of standardized policies governing mental healthcare delivery, including service accreditation requirements and provider reimbursement structures, not only restricts the range of quality services available to patients but also reduces providers’ capacity to deliver integrated, coordinated care. In line with Raghavan et al. (47), sustainable mental healthcare requires a multifaceted policy approach that integrates financial, regulatory, and organizational supports to overcome these systemic barriers and ensure continuity and quality of care.

### 4.1 Implications of findings

Improving mental healthcare in Singapore requires coordinated action across individual, organizational, and policy levels. At the individual and community level, strategies to reduce stigma and promote early intervention are critical starting points. To reduce stigma and promote early intervention, mental health education should be integrated into school curricula and professional training for teachers, employers, and other key community stakeholders. Participants also suggested that community-wide awareness campaigns could help to normalize discussions around mental health and encourage individuals to seek help sooner, thus alleviating pressures on more specialized care services. Additionally, improving awareness of community-based services, such as counseling services at family service centers, was highlighted. Establishing peer support systems, where individuals with lived experience provide ongoing emotional support (48), can help bridge the gap for those waiting for professional care. Strengthening informal support networks by empowering families, friends, and community organizations to provide immediate assistance would also reduce the burden on formal mental healthcare services, ensuring timely support for those in need.

At the organizational level, fragmented service delivery highlights the need for clearer inter-organizational communication and coordination. Standardizing referral processes and creating seamless communication channels, such as linking GPs more systematically with allied health teams like Community Intervention Teams (COMIT) and Community Outreach Teams (CREST) (49, 50), will improve patients’ transition between services. Importantly, integrating information systems across healthcare and social care sectors enables real-time access to patient data, supporting care continuity and facilitating timely, coordinated interventions. Strategic workforce development is also essential. This includes addressing skill gaps, improving staff retention, ensuring remuneration aligns with caseload demands, and proactively supporting providers’ mental health to prevent burnout.

At the macro level, expanding insurance coverage to include common mental health conditions like depression and anxiety would facilitate broader access to mental healthcare (51, 52). Private insurance should also be extended to include more comprehensive mental health coverage, moving beyond specific target populations (53). Reforming reimbursement structures is crucial to incentivize private sector GPs to actively engage in integrated care models, while promoting the development of mental health interventions that support individuals at various life stages and with different conditions. Additionally, adjustments should be made to allow lower-income individuals seeking care from private sector GPs to access state-sponsored assistance, thereby expanding options for first-touchpoint care (54).

Strengthening regulations for private sector mental healthcare is essential for protecting patient rights and ensuring service quality. These efforts could include more channels for redress which would allow patients to voice concerns and seek resolution, as well as enhancing oversight mechanisms to ensure consistent service delivery and protect users from potential harm (55, 56). Moreover, participants suggested the need to tailor data protection laws for secure data sharing across providers and for data rights to reside with patients. This tailored data policy would allow patients to decide which providers can access their information to facilitate care coordination. Finally, fostering flexible collaborations between public and private sectors through joint case discussion platforms and cross-sector collaboration platforms – including healthcare, education, and social services – will be key to supporting an integrated and responsive mental healthcare system.

### 4.2 Strengths and limitations of project

Among our project’s strengths, one was the inclusion of views of providers from a wide variety of professional backgrounds, facilitating a comprehensive understanding of challenges faced from various roles and both public and private sectors. Another strength was the use of the RE-AIM and SELFIE frameworks. The RE-AIM framework facilitated a systematic investigation of barriers to implementing accessible, effective, and sustainable mental healthcare. The SELFIE framework facilitated a comprehensive understanding of barriers at the micro, meso, and macro levels, and how these barriers collectively contributed to care gaps and actionable recommendations. Nevertheless, there were a few limitations, including the lack of service users’ perspectives, due to the scope of the project. Another limitation was the lack of perspectives from caregivers (57) and employers of individuals with mental health needs (58). Future projects could thus triangulate views of service providers, patients, caregivers, and employers, for additional insights.

## 5 Conclusion

Findings from this qualitative service evaluation project provided insights into barriers to accessible, effective, and sustainable mental healthcare in Singapore that is based on a biopsychosocial model of care leveraging community-based providers such as GPs as first touchpoints. These barriers included limited systemic integration of services and information systems, constraints due to referral routes and lengthy forms, limited public sector capacity, private sector GPs’ viability issues, and lack of regulation of private sector AHPs. Our findings also affirmed the importance of addressing socio-cultural barriers of stigma and limited mental health literacy levels to improve care gaps. More needs to be done in Singapore, as well as other countries facing similar issues, to strengthen regulation of private sector services, further develop capacity, tap on social networks and peer support groups, improve service integration, increase mental health literacy levels and reduce stigma, to achieve more accessible and effective mental healthcare provision to all individuals with mental health needs.

## Data Availability

The participants of this study did not give written consent for their data to be shared publicly, so due to the sensitive nature of the project, supporting data is not available.
