# Supplementary material for: Barriers underlying care gaps in Singapore’s mental health landscape and suggestions for improvement from service providers’ perspectives: a qualitative approach
Source: Front Public Health. 2025 May 19;13:1527521. doi: 10.3389/fpubh.2025.1527521 (PMC12127186; doi:10.3389/fpubh.2025.1527521)
Supplement: Supplementary file 1 [file Supplementary_file_1.pdf]

SEMI-STRUCTURED INTERVIEW GUIDE

[For Interviewer]

Thank you for taking the time to join this interview. I am (name of interviewer) from the Centre of Population Health Research and Implementation in SingHealth and we are here to understand the local mental health landscape from your perspective and professional experiences in the field. This is a joint study with Sheares Healthcare which is a wholly owned operating company of Temasek, with a vision to transform healthcare in Asia.

This interview will take around 1 hour of your time. Please be assured that your information will be kept strictly confidential and will only be used for research purposes in this study. Any findings reported will not contain identifiable information, so do speak freely as there are no right or wrong answers. We would love to hear your opinions regarding the landscape of mental healthcare in Singapore. At any point if you feel uncomfortable and prefer not to respond to certain questions or experience fatigue during the interview, please let us know.

At this point, do you have any questions for me?

[If no] May I have your permission to record this interview?

A. INTRODUCTION

1. Could I ask you for an introduction of yourself?
   - Your position within [*name of participant's organisation*]
   - Your roles and responsibilities within [*name of participant's organisation*]
   - How long have you been part of [*name of participant's organisation*]?
   - How long have you been involved in the local mental health landscape?

B. REACH

2. What is the general profile of individuals seeking mental healthcare services at [name of participant's organisation]?
   - Now we would like to ask about the needs, preferences and priorities of these individuals.
     - What are their demographic profiles?
     - What are the key mental healthcare needs and preferences of these individuals, including whether they have a preference for in person or digital services, or a hybrid of both (digital mental health services refer to the delivery of effective mental health services using the internet and related technologies, and examples include video consultations with clinicians and mental health professionals, cognitive training via mobile, web-based or computer devices or virtual reality etc)
     - What is the availability of their social networks?
     - How are patients financing the mental health services that they receive (self-paying/subsidized/insured/corporate sponsored etc)?

        - how do patients receive information about services?

3. What are the available mental health services for these clients/patients at your organisation? In your opinion, how do you think these services meet the needs for the segment(s) of population that you provide care for?
        - Are these services in place as part of your organisation's routine programmes and how have the services been integrated into your organisation's policies?

4. What factors are taken into consideration when deciding on the referral of appropriate mental healthcare services to clients?
        - Are there 'eligibility gaps' among systems, services, age categories, or entry criteria? (Probe: Are the entry criteria for mental health services too broad/narrow?)
        - Are there any underserved population segments that should be brought into attention? Why do you think that the current mental healthcare services are not reaching this/these population segment(s)? (Probe: eligibility gaps, health-seeking behaviours, cultural mores, availability of services etc)
        - What are the needs and preferences of this/these population segment(s)? What improvements to current services/ additional services would you suggest to address the needs and preferences of this/these underserved segment(s)?

5. How accessible do you think local mental healthcare services are?
    - How is the process like for providers to navigate available mental healthcare services and resources in Singapore?
        - Where (and how) can providers access information on available mental healthcare services and resources?
        - How well-informed do you think the providers like yourself are about available mental healthcare services and resources in the health and social care sectors?

C. EFFECTIVENESS

6. How effective do you think the existing services are in supporting the mental healthcare needs of the different population segments in Singapore? Why is that so?
    - What are the measures to evaluate the effectiveness of services?
    - Are there specific population segments for whom digital mental health services and resources have been or might be particularly helpful and if so, which population segments are they and in what ways?

D. ADOPTION

7. What is the current mental healthcare model adopted by [*name of participant's organisation*]?
    - How does this mental healthcare model help with mental healthcare delivery and practice by providers in [*name of participant's organisation*]?
    - Do you think the current care model can also meet the needs and care required by the aforementioned underserved population segment(s)? Why is that so?
        - [If no] What could be appropriate adjustments to ensure appropriate mental health care is also provided to the underserved segment (s)? (*Probe:* Do you think the model of care should be team-based/led by specialists? Would you suggest using digital services or AI as an enabler?)

8. In your opinion, what would be the ideal roles for the following stakeholders in mental healthcare?
    - GPs (*Probe:* e.g. referred from specialists for monitoring of stable cases)
    - Allied health professionals such as psychologists, counsellors and social workers
    - Nurses (*Probe:* e.g. what do you think about the feasibility of nurse counsellors working with GPs to support patients in managing chronic mental health conditions?)

E. IMPLEMENTATION

9. Is the current mental healthcare system well-integrated to the overall local healthcare system to provide care for individuals at different stages of their mental healthcare journey?
    - In your opinion, how do nationwide and sector wide policies facilitate integration of services and systems?
    - How is the relationship between mental healthcare providers in Singapore, with regards to patient management and care coordination?
        - Is the transition from one service to another smooth and monitored throughout the care process? What facilitates/challenges the continuity of care?
        - Would you have any suggestions to improve coordination between providers to facilitate integrated care provision?

10. Is there currently a shared information system used among providers to facilitate information exchange, track progress and decide on future care plans for patients?
    - [if yes] How would providers, clients and their informal caregivers benefit from such a system?
    - [If such a shared information system is in place]What are the implications on access to information, data ownership and protection?
        - What mental healthcare privacy and data protection legislation are/should be in place to address the implications?
11. What are the digital mental health services that your organisation is offering or would like to offer? For relatively new modes of service delivery, such as digital mental health services/provision of online mental health resources, what are some of the facilitators and barriers to implementation experienced by yourself and your colleagues?

12. What are some other pain points for yourself and your patients/clients that you would like to share with us? (*Probe:* e.g. for patients: wait time in public / VWO sector; for providers: caseload per day)

F. MAINTENANCE

13. What practices and skills do professionals in your field consider important and necessary in providing effective mental healthcare?
    - How does the current professional curriculum prepare one to undertake work in mental healthcare?
    - What platform(s) do you know of for mental healthcare professionals to further develop their professional knowledge and skills and/or engage in knowledge sharing and discuss best practices?
    - How adequate are the existing professional training programmes and knowledge sharing platforms in facilitating provision of appropriate mental healthcare to the aforementioned underserved population segment(s)?

14. Do you think there is currently sufficient mental healthcare capacity to cater to the mental health needs in Singapore?
    - What are existing strategies/plans to improve the local capacity to provide mental healthcare services?
    - In the long run, how can we ensure sufficient capacity to meet the mental health needs of Singaporeans? (*Probe:* Continuous professional education and development, addressing workforce-demography match and changes in type of care provisions, attracting more talents into the field and sustaining their passion in the field, consideration of new professional roles)

15. What are some developments in the local mental healthcare system that you foresee in the near future? What are suggestions for improvement of the mental healthcare landscape at the organisational and/or policy level?
    - Are there national/regional policies and action plans on mental healthcare development that you are aware of?
    - Are there any recent developments or policy considerations regarding mental health services that harness digital technology that you are aware of?
    - Are the current payment systems/schemes for mental healthcare providers appropriate? How can organisations be financially supported to ensure sustainable commitment to providing quality mental healthcare for different population segments? (*Probe:* e.g. through subsidies and insurance)
    - How are providers working with employers to support mental healthcare needs of employed Singaporeans?

G. BROADER SOCIO-CULTURAL ISSUES AFFECTING MENTAL HEALTH CARE

16. What are some community-level barriers that affect timely use of mental healthcare services by individuals in Singapore, especially among underserved population segment(s) (e.g. stigma, lack of mental health awareness and literacy)?
    - What can be done to encourage the underserved population segment(s) to seek the mental healthcare services they require?

<u>COVID-19</u>

17. How has COVID-19 impacted the mental health of Singaporeans (in particular, the segment(s) of population that you provide care for)?
    - How are appropriate support/resources provided to meet the mental health needs during the pandemic? How has technology played a part in the adaptation of services/service delivery modes during the pandemic?
    - Did you notice any differences with regards to the segment(s) of population seeking mental healthcare services during COVID-19?
        - Were there unexpected segment(s) of the population seeking mental healthcare services during the pandemic? Who are they? Why do they require mental healthcare services?
    - Post-pandemic, would there be transformation to the mental healthcare landscape in Singapore? How are we coping with the transformation?

H. FINAL COMMENTS

18. Before we end this session, is there anything else that we have not touched on, but you think it is important for us to know about the mental health landscape in Singapore?

Thank you, once again, for taking the time to participate in this interview. We have learned a lot about the local mental health landscape from your perspective. I will be stopping the recording for now.
